# Prevalence of Using Complementary and Alternative Medicine in Children and Its Related Factors in East Iran

**Published:** 2013-11-22

**Authors:** Azita Fesharakinia, Mohammadreza Abedini

**Affiliations:** 1 Department of Pediatrics; 2 Department of Physiology and Pharmacology, Complementary Medicine Research Center, Birjand University of Medical Sciences, Birjand, Iran

**Keywords:** Complementary Medicine, Alternative Medicine, Unconventional Therapy, Children

## Abstract

***Objective:*** Recently application of complementary and alternative medicine (CAM) is increasing in children worldwide. The present study was conducted to determine the prevalence, related factors, types, the sources of information and knowledge of mothers for the possible side effects.

***Methods:*** This descriptive and analytical study carried out within three months from April-June 2012 through oral interviews and questionnaires with 300 mothers of children referred to pediatric clinic in Vali-Asr Hospital, Birjand (center of South Khorasan province: East of Iran).

***Findings*** ***:*** 35.6% of mothers had used CAM as medication at least once for their children during the last year. There was a significant and direct correlation between using CAM for children with increased maternal age, decreased level of mother’s education, mother being as a housewife and having more than two children. Most (93.3%) common treatments included medicinal herbs, oil rub (26.6 %) and prayer therapy (25.7%). Relative (72%) and neighbors (50%) were the most sources for mother’s information while physicians consist only 2% of the information source. Only 1.3% of mothers knew that CAM may also exert some side effects.

***Conclusion:*** Considering the fact that about one third of mothers used CAM modalities, physicians were the least maternal source of CAM information, nearly all mothers were unaware of the side effects of CAM It is recommended that physicians should learn about the CAM to enable them for providing information to parents regarding its benefits and disadvantages. It is also highly recommended to enhance community knowledge about the proper use of different kinds of CAM.

## Introduction

Complementary and alternative Medicine (CAM) refers to a wide range of views and actions that have not been precisely defined in current standard medicine. It is neither taught in conventional medical schools, nor applied in hospitals. CAM includes various methods for disease treatment or prevention with a different efficacy approach from traditional medicine.

 Various types of CAM are quite diverse in different parts of the world and influenced by culture, history, level of education and individual interests. Countries such as China, South Korea and Vietnam have accepted complementary medicine into their health systems^[^^1^^]^.

 In Africa, about 85% of people tend to use alternative medicine to treat various diseases^[^^2^^]^. CAM is also applied at different extents in developed countries. American academy of pediatrics (AAP) reported that 20-40% of healthy and 50% of children suffering from chronic diseases receive CAM therapies^[^^3^^]^. Studies conducted in different countries suggest that variety and rate of CAM for children are quite different^[^^4^^-^^9^^]^.

 Due to increasing rate of CAM application in children worldwide, it is highly recommended to recognize its worth appropriately among people. Therefore, through increasing people’s knowledge in this context, and appropriate application of it for patients, one would be able to promote the patient care for children worldwide. This research project was conducted under supervision of the Research Council of Birjand University of Medical Sciences on mothers to determine the prevalence, related factors, types, the information sources and knowledge for the possible side effects.

## Subjects and Methods

The present study conducted on the mothers of 300 children who were admitted to the general pediatric clinic in Vali-Asr Hospital, Birjand during spring (April –June) 2012. This hospital, as the main hospital in town, accepts all referrals from different strata of the society.

 To avoid recall bias, initially the project objective has been explained for mothers. It was also mentioned that this is just a research project, the mother’s and child’s names will be not recorded. Moreover, the participation is done completely voluntarily. Then a trained nurse interviewed the mothers who were interested to participate, and a questionnaire was completed. First of all the nurse gave necessary information about CAM and its variants including medicinal herbs, prayer therapy, acupuncture, unction, cupping therapy, leech therapy, hydrotherapy, etc. The questionnaire included demographics information for children (age and sex) and for mothers (age, education level, employment status and number of children), the use of this kind of therapy at least once during the last year for their children, divers types of CAM used, the sources of maternal information and knowledge for the possible side effects of CAM.

 The face and content validity of the questionnaire were approved by consulting with several expert faculty members and its reliability was determined after completing 30 questionnaires and calculating the Cronbach's alpha coefficient. Data entered into the SPSS software (version 17) and were analyzed by descriptive statistics (mean and frequency) and analytical statistics (Chi-square test). *P* value less than 0.05 was considered as significant.

## Findings

All 300 mothers agreed to participate in the study. Median age of mothers and children was 31.65±4.74 and 3.24±2.60 years, respectively. 66% of children were less than three years old and 52% were boys. The majority of mothers (87%) were housewives and most of them (61.2%) had one or two children. 35.6% of mothers had used CAM.

 There was a significant and direct correlation between using CAM with increased maternal age, decreased level of mother’s education, mother being as a housewife and having more than two children (Table 1). Regarding the type of CAM which was used, 105 mothers responded to this section. The most common treatment was medicinal herbs (93.3%), oil rub (26.6%) and prayer therapy (25.7%).

 In terms of the information source for CAM, 196 mothers responded to this section. Family (72%) and neighbors (50%) were the most, while physicians consisted the least (2%) source of the information. Since many of the mothers had selected more than one response about types and information source, the overall percentage in these two sections was over 100%.

 Only 1.3% of total mothers (300 subjects) knew that complementary medicine may also exert side effects at some extent.

**Table 1:** CAM usage for children based on children and mothers demographic characteristics

**Use of CAM in the treatment of children**		**No**	**Yes**	**Total**	***P*** **-Value**
**Child sex**	Girl	94 (65.3%)	50 (34.7%)	144 (100%)	0.74
	Boy	99 (63.5%)	57 (36.5%)	156 (100%)	
**Child age**	< 3 years	132 (66.7%)	66 (33.3%)	198 (100%)	0.24
	≥ 3 years	61 (59.8%)	41 (40.2%)	102 (100%)	
**Number of children**	≤ 2 child	137 (74.9%)	46 (25.1%)	183 (100%)	<0.001*
	> 2 child	56 (47.9%)	61 (52.1%)	117 (100%)	
**Maternal employment**	Housewife	157 (60.2%)	104 (39.8%)	261 (100%)	<0.001*
	Employed	36 (92.3%)	3 (7.7%)	39 (100%)	
**Maternal level of education**	Illiterate	3 (14.3%)	18 (85.7%)	21 (100%)	<0.001*
	Primary	35 (37.6%)	58 (62.4%)	93 (100%)	
	Middle school	61 (76.2%)	19 (23.8%)	80 (100%)	
	Diploma	80 (95.1%)	6 (4.9%)	86 (100%)	
	University	14 (70%)	6 (30%)	20 (100%)	
**Maternal age (years)**	20 to 30	106 (71.1%)	43 (28.9%)	149 (100%)	0.03*
	30 to 40	80 (58.8%)	56 (41.2%)	136 (100%)	
	≥ 40	7 (46.7%)	8 (53.3%)	15 (100%)	

## Discussion

Due to increasing rate of CAM application in children worldwide, determination of the rate of CAM application and the factors influencing the usage of it is very important. In the present study, about one-third of mothers have used CAM for their children. This rate was 58.6% and 57% in Turkey^[^^4^^,^^5^^]^; 33% at St. Louis^[^^6^^]^; 31% in Nigeria^[^^7^^]^ and 12% in Pittsburgh^[^^10^^]^.

 Unlike other studies^[^^4^^,^^5^^,^^9^^]^ that higher maternal education levels were usually associated with more CAM application, in the present study lower level of maternal education was correlated with higher CAM usage for their children. This could be due to the fact that educated mothers in Birjand have opposite views about using this type of medicine.

 In this study, there was a significant increase in the use of CAM in children with mother being as housewife, but in a study in Karachi^[^^9^^]^, being employed or working as a housewife had no significant influence on the usage of CAM. Significant correlation with mothers being a house wife may be related to this fact that these mothers have low education levels.

 In this study and In Cardiff^[^^11^^]^ and Bass^[^^12^^]^ studies, there was no significant association between children's age and gender with CAM usage. 

 Honey followed by herbal tea in Karachi^[^^9^^]^, biological products in Nigeria^[^^7^^]^, herbal remedies (41%) and prayer therapy (37%) in Michigan^[^^8^^]^ and herbal medicine in Turky^[^^4^^,^^5^^]^, were the most used CAM. In the present study, the most common treatment was herbal medicine. It is due to its special place among the people of this region which is a rich region in terms of herbal medicine. 

 Family and neighbors in the present, Turkey^[^^4^^]^ and Karachi^[^^9^^]^ studies and friends and neighbors in Nigerian study^[^^7^^]^ were the most sources of maternal information for using CAM. In all of these studies physicians had no important role for giving information to people. Since information from family and neighbors may be more confusing rather than it is helpful, people reasonably must receive information and guidance on the efficacy and side effects of CAM from their doctors.

 In the present study, only 1.3% of mothers were aware of the possible side effects of this treatment. Herbal medicine was the most common treatment in the present study. Since these herbs may have some side effects and interaction with modern medication, it is recommended that pediatricians ask parents about CAM using, warn them regarding the possible risk of using these drugs, and educate them to use the appropriate form.

 This study has several limitations. Information gathered just in one clinic and that is a hospital clinic which runs with modern medicine. Perhaps, if the study has been performed in a traditional medical center, it would have led to quite different results. Mothers, who refer to a modern medical clinic, may be afraid to be blamed by doctors if they give the actual information about the usage of CAM. This is a native and regional study and the results might be different from another city in a different geographical area.

## Conclusion

Considering the fact that about one third of mothers used CAM modalities, physicians were the least maternal source of CAM information, nearly all mothers were unaware of the side effects of CAM. It is recommended that physicians learn about the CAM to enable them providing information to parents regarding its benefits and disadvantages. It is also highly recommended to promote community knowledge about the proper use of different kinds of CAM.

## References

[1] WHO WHO launches the first global strategy on traditional and alternative medicine. online 2002 (cited 2008 Oct 20).

[2] Kofi-Tsekpo M (2004). Institutionalization of African traditional medicine in health care system in Africa. Afr J Health Sci.

[3] Kemper KJ, Vohra S, Walls R, American Academy of Pediatrics (2008). The use of complementary and alternative medicine in pediatrics. Pediatrics.

[4] Araz N, Bulbul S (2011). Use of complementary and alternative medicine in a pediatric population in south Turkey. Clin Invests Med.

[5] Ozturk C, karayagiz G (2008). Exploration of the use of complementary and alternative medicine among Turkish children. J Clin Nurs.

[6] Loman DG (2003). The use of complementary and alternative health care practices among children. J Pediatr Health Care.

[7] Oshikiya KA, Senbanjo IO, Ngokanma OF (2008). Use of complementary and alternative medicines for children with chronic health conditions in lafos, Nigeria. BMC Complement Altern Med.

[8] Sawni-Sikand A, Schubiner H, Thomas RL (2002). The use of complementary/alternative therapies among children in primary care pediatrics. Ambul Pediatr.

[9] Ashraf S, Rahman AJ, Satwani H (2010). Trend of complementary therapies in pediatric age group. J Pak Med Assoc.

[10] Pitetti R, Singh S, Hornyak D (2001). Complementary and alternative medicine use in children. Pediatr Emerg Care.

[11] Karadeniz C, Pinarli FG, Oguz A (2007). Complementary/alternative medicine use in a pediatric oncology unit in Turkey. Pediatr Blood Cancer.

[12] Crawford NW, Cincotta DR, Lim A (2006). A cross-sectional survey of complementary and alternative medicine use by children and adolescents attending the university hospital of Wales. BMC Complement Med.

